# Exploring the Interplay between Cellular Senescence, Immunity, and Fibrosing Interstitial Lung Diseases: Challenges and Opportunities

**DOI:** 10.3390/ijms25147554

**Published:** 2024-07-10

**Authors:** Fernanda Hernandez-Gonzalez, Federico Pietrocola, Paolo Cameli, Elena Bargagli, Sergio Prieto-González, Tamara Cruz, Nuria Mendoza, Mauricio Rojas, Manuel Serrano, Alvar Agustí, Rosa Faner, Jose A. Gómez-Puerta, Jacobo Sellares

**Affiliations:** 1 Department of Respiratory Medicine, Respiratory Institute, Hospital Clinic Barcelona, 08036 Barcelona, Spain; aagusti@clinic.cat (A.A.); sellares@clinic.cat (J.S.); 2 Instituto de Investigaciones Biomédicas August Pi i Sunyer (IDIBAPS), 08036 Barcelona, Spain; sprieto@clinic.cat (S.P.-G.); cruz@recerca.clinic.cat (T.C.); nmendoza@recerca.clinic.cat (N.M.); rfaner@recerca.clinic.cat (R.F.); 3 Faculty of Medicine and Health Sciences, University of Barcelona, 08036 Barcelona, Spain; 4 Department of Cell and Molecular Biology, Karolinska Institutet, 17165 Solna, Sweden; federico.pietrocola@ki.se; 5 Respiratory Diseases Unit, Department of Medical and Surgical Sciences & Neuro-Sciences, University of Siena, 53100 Siena, Italy; paolo.cameli@unisi.it (P.C.); bargagli2@gmail.com (E.B.); 6 Vasculitis Research Unit, Department of Autoimmune Diseases, Hospital Clinic Barcelona, 08036 Barcelona, Spain; 7 Centro Investigación Biomédica en Red Enfermedades Respiratorias (CIBERES), 08036 Barcelona, Spain; 8 Division of Pulmonary, Allergy and Critical Care Medicine, Department of Medicine, The Ohio State University Wexner Medical Center, Columbus, OH 43210, USA; mauricio.rojas@osumc.edu; 9 Cambridge Institute of Science, Altos Labs, Cambridge CB21 6GP, UK; mserrano@altoslabs.com; 10 Biomedicine Department, University of Barcelona, 08036 Barcelona, Spain; 11 Rheumatology Department, Hospital Clinic Barcelona, 08036 Barcelona, Spain

**Keywords:** lung fibrosis, cellular senescence, immunosenescence

## Abstract

Fibrosing interstitial lung diseases (ILDs) are characterized by the gradual and irreversible accumulation of scar tissue in the lung parenchyma. The role of the immune response in the pathogenesis of pulmonary fibrosis remains unclear. In recent years, substantial advancements have been made in our comprehension of the pathobiology driving fibrosing ILDs, particularly concerning various age-related cellular disturbances and immune mechanisms believed to contribute to an inadequate response to stress and increased susceptibility to lung fibrosis. Emerging studies emphasize cellular senescence as a key mechanism implicated in the pathobiology of age-related diseases, including pulmonary fibrosis. Cellular senescence, marked by antagonistic pleiotropy, and the complex interplay with immunity, are pivotal in comprehending many aspects of lung fibrosis. Here, we review progress in novel concepts in cellular senescence, its association with the dysregulation of the immune response, and the evidence underlining its detrimental role in fibrosing ILDs.

## 1. Introduction

The primary predictor for many diseases is chronological age, which contributes significantly to morbidity, mortality, and healthcare costs [1]. Accelerated aging has been described as the origin of several chronic diseases, including interstitial lung diseases (ILDs) [2]. Idiopathic pulmonary fibrosis (IPF), the most common and fatal ILD, leads to progressive lung architecture destruction and irreversible respiratory failure [3,4]. However, within the spectrum of fibrosing ILDs there is a subset of patients with fibrosing ILD of known or unknown etiology other than IPF who exhibit a similar clinical course and prognosis, named progressive pulmonary fibrosis (PPF).

Recent research indicates that aging, cellular senescence and disturbances of immune mechanisms may contribute to an inadequate response to stress and increased susceptibility to lung fibrosis. The incomplete understanding of the underlying biology of fibrosing ILDs and its heterogeneity directs scientific research toward developing biomarkers and therapeutic tools to enhance the life expectancy of affected patients.

Below, we review recent advances in comprehending emerging concepts in cellular senescence, its influence on immune response dysregulation and the evidence emphasizing its detrimental role in lung fibrosis.

## 2. Brief Overview of Lung Fibrosis Pathogenesis

In fibrosing ILDs, fibrosis and wound healing are closely connected processes, orchestrated by a sequence of events including injury, inflammation, fibroblast proliferation and migration, matrix deposition and remodeling. The pathological accumulation of fibrotic tissue is a process marked by intricate interactions among epithelial cells, fibroblasts, immune cells and endothelial injury [5,6,7]. Numerous interconnected wound-healing pathways are triggered to facilitate the lung tissue repair, turnover and adaptation in response to lung injury. However, aged cells are unable to repair lung damage due to impaired cellular maintenance and regeneration mechanisms.

In this context, recent investigations underscore the fundamental involvement of cellular senescence in the pathogenesis of age-related diseases, such as pulmonary fibrosis. Cellular senescence, a well-known hallmark of aging, is a condition characterized by essentially irreversible cell cycle arrest upon unrepairable damage while viability is preserved. Senescent cells exhibit two common phenotypic attributes, including an incapacity for proliferation and the production of an extensive pro-fibrotic and pro-inflammatory secretome (referred to as SASP, senescence-associated secretory phenotype). Senescent cells elicit ambivalent effects, which may vary depending on the intensity of the trigger, cell type, disease stage, or age, reflecting the concept of antagonistic pleiotropy [8,9,10]. Cellular senescence typically advances in harmony with many other aging mechanisms, so-called “hallmarks of aging” (telomere attrition, stem cell exhaustion, loss of proteostasis, disabled macroautophagy, deregulated nutrient-sensing, mitochondrial dysfunction and altered communication) and “meta-hallmarks of aging” (genomic instability, epigenetic alteration, chronic inflammation and dysbiosis) [10,11]. Thus, the progressive accumulation of senescent cells and impaired immunosurveillance contribute to the pathogenesis of numerous diseases, age-related dysfunction and the decline of resilience. In the lung, there is a relatively straightforward relationship with several environmental cell injury factors, so the senescent cell fate can be triggered by a number of dangerous stressors that intercept various effector pathways, resulting in DNA damage, oxidative stress, endoplasmatic reticulum stress or mitochondrial damage inducing the activation of the p53/p21CIP1/WAF1 and p16INK4a/retinoblastoma protein axes, leading to cell senescence and to the development of a pro-fibrotic secretome [11]. This characteristic could also elucidate the vulnerability of the lung to increase in senescence-inducing conditions contributing to pulmonary fibrosis. In this regard, studies have shown that senescent cells alone can induce lung tissue dysfunction associated with aging, reinforcing the suggested causal link between senescent cell accumulation, aging, and disease [12,13].

Furthermore, one plausible explanation for the accumulation of senescent cells in the aged lung is an altered immune system capacity to eliminate these cells, which can either be associated with structural and functional changes of an aging immune system (termed “immunosenescence”) or with a deficient immune response skewed to tolerance [14]. Either situation leads to less efficient clearance of senescent cells, thereby facilitating the development of lung fibrosis. Therefore, understanding these immune-related adaptations in lung fibrosis development requires initiatives aimed at identifying signaling pathways.

## 3. Immunosenescence and Senescent Cell Immunosurveillance

It remains crucial to comprehend the interplay between aging, immunosenescence and pulmonary conditions like pulmonary fibrosis for advancing research. Immunosenescence refers to the decline in cellular and adaptive immunity due to age-related changes. It encompasses several essential components: remodeling of the immune organs, age-associated impaired innate and adaptive immune functions, impaired vaccine responses and increased susceptibility to infections and malignancies [14,15,16,17]. Moreover, immune resilience, also termed immunocompetence, which refers to the capability to maintain or quickly recover immune functions that aid in disease resistance and manage immune response during injury or other sources of stress, is a characteristic observed throughout the age range [18]. Thus, this trait may be associated with a particular balance between immunocompetence and immunosenescence that correlates with positive health outcomes dependent on immunity. This observation prompts exploration of age-related immunological changes, considering a blend of resilience and restructuring versus immunological maladaptation [19,20]. Advocating for a novel approach, this emerging field targets aging as the primary risk factor, recognizing individual variations in the aging process. Theories such as autoimmunity, immunodeficiency, immune dysregulation or a combination of these factors have been proposed to explain this physiological phenomenon. Moreover, oxidative stress is a significant factor in the process of immunosenescence, affecting both innate and adaptive immunity by accelerating telomere shortening and mitotic clock values through alteration of DNA methylation, although the exact mechanisms remain unclear [21].

Noteworthy, an interplay between the immune system and senescent cells exists. There are now compelling proof-of-concept studies supporting the primary role of innate immune cells in removing senescent cells [22,23,24,25]. Senescent cells interact with both the innate and adaptive components of the host immune system. The recognition and clearance of senescent cells involve both humoral and cellular components of adaptive immunity. As previously noted in the context of neoplastic malignancies, the function of host leukocytes as “immune senolytics” also extends to non-oncological conditions [22]. Thus, senescent cells can disrupt the microenvironment by attracting various immunosuppressive immune subsets and might exhibit susceptibility to antibody-dependent cell-mediated cytotoxicity after generating natural antibodies targeting surface antigens [26,27].

On the other hand, senescent cells may also escape immune surveillance through the expression of senescence-associated immune checkpoints, such as calreticulin, CD47, or non-classical MHC-I molecules, which deactivate cytotoxic immune cells and determine the susceptibility of target cells to phagocytosis [28,29,30]. The enduring existence of senescent cells in aged or damaged tissues entails immune-evading strategies coordinated by these resilient variants to avoid immune intervention.

Furthermore, SASP mediators and extracellular vesicles contribute to the senescence-driven cellular response, impacting tissue functions in a non-cell autonomous manner. Via autocrine or paracrine secretion, there is a chemoattraction of immune cells in charge of senescent cell immunosurveillance that might modulate their function [31]. Once the threshold of senescent cell burden is exceeded, continued escalation in pro-apoptotic and pro-inflammatory senescent cell burden can exacerbate tissue damage, thereby contributing to the onset or progression of diverse diseases and age-related disorders. This process of immune dysregulation fosters a feed-forward loop, further amplifying senescent cell accumulation [19,32].

Interestingly, the role of exosomes in pulmonary fibrosis progression and cellular senescence has grown significantly [31,33]. Exosomes, nanoscale membrane-bound vesicles released by nearly all cell types, transport various biomolecules (nucleic acids, lipids and proteins) and are key mediators of cell-to-cell communication [34]. They are vital for antigen presentation, immune response, immunomodulation, inflammation, and cellular phenotypic transformation, playing a key role in lung fibrosis [35]. For instance, let-7d-5p was shown to be related to cell cycle regulation and senescence and its downregulation in pulmonary fibrosis lungs noted [36]. Its expression also decreased in patients with acute exacerbation and stable pulmonary fibrosis compared to controls [35]. However, the specific cellular targets of miRNAs in pulmonary fibrosis and their connection to cellular senescence remain unclear, so further investigation into their mechanisms is needed.

Moreover, extensive research has identified the complement system and unbalanced SASP as two main inducers of lung fibrosis [37]. Thus, understanding the different pathways and cytokines that regulate SASP may enable us to identify potential targets for pharmacological inhibition.

## 4. Senescence-Related Immune Cells Subsets in Fibrosing Interstitial Lung Diseases

**Figure 1:**
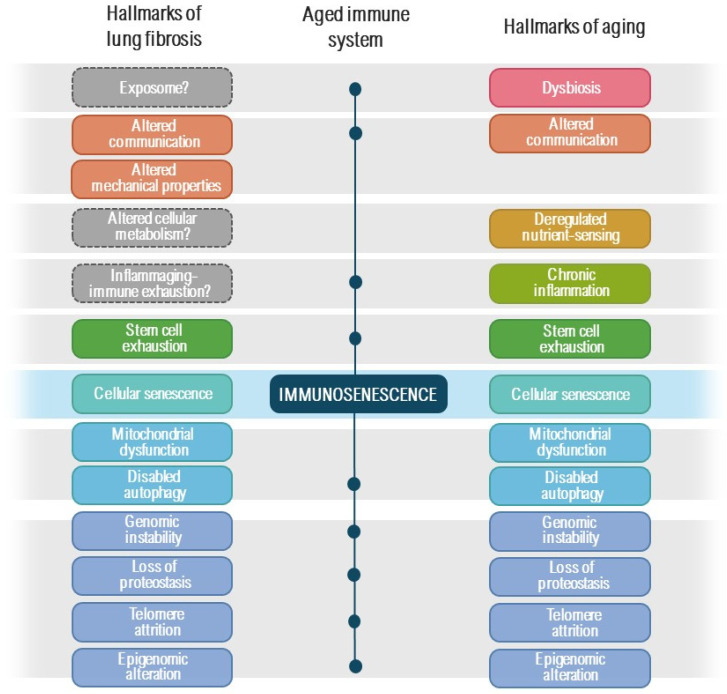
Overview of the hallmarks of aging, lung fibrosis, and their similarities with the main pathways of immunosenescence. The diagram illustrates the points of overlap between the hallmarks of aging (on the **right**) and lung fibrosis (on the **left**), suggesting that many of these factors likely contribute to immunosenescence and lung fibrosis.

The intricate interaction of environmental and genetic elements, age-associated mechanisms, and epigenetic modifications results in significant age-related changes in alveolar epithelial cells, immune cells and fibroblasts within fibrotic lungs [38,39]. The human respiratory system requires a carefully controlled response to constant exposure to various challenges during life due to invading pathogens and foreign materials, the so-called “exposome”. The primary alterations in the aging adaptive immune system are observed within the T cell compartment [40,41]. Collectively, decreased thymic export, increased antigen exposure, and dysregulation in homeostatic maintenance mechanisms in both the naïve and memory T cell populations contribute to the overall decline in repertoire diversity with age. In this context, the senescent state of the immune system may weaken the immune response to these stimuli. The role of the immune response in the pathogenesis of pulmonary fibrosis remains unclear. However, studies suggest abnormalities in circulating immune cells, particularly in cytotoxic cells, among IPF and PPF patients, indicating an aged and exhausted immunophenotype [42,43]. Overall, there exists a striking similarity between the hallmarks of aging and those of lung fibrosis, as well as of immunosenescence (Figure 1).

### 4.1. Stem Cell Exhaustion

One of the most important hallmarks of aging contributing to the lung fibrosis process is the decline in adult epithelial stem cell reserves. Age-related quantitative and qualitative changes secondary to age-associated stem cell exhaustion have been observed in various progenitor populations in the lung. For instance, basal and club cells decrease with age, while alveolar epithelial type 2 cells (AEC2s) maintain their quantity but show deficits in self-renewal and differentiation capacity [6]. AEC2s are immunologically active as they can produce cytokines that impact neighboring immune cells [5]. The SASP effect of senescent AEC2s is harmful, potentially inducing a variety of pro-fibrotic mediators that trigger an abnormal wound-healing response [44]. Recent research indicates that NF-κB and CEBPB (CCAAT/enhancer-binding protein-β) are crucial in inducing SASP factors, particularly by initiating an autocrine feedback loop involving IL-6 and IL-8 [45]. Furthermore, telomere dysfunction can impede the clearance of senescent cells by impairing immune function, particularly in individuals with short telomeres and IPF [46]. Therefore, the depletion of lung epithelial stem cells disrupts the normal interaction between epithelial cells and the lung immune system. Additionally, deletion of Ercc1 in hematopoietic stem cells and deficiency in perforin lead to premature immunosenescence and multi-organ aging, indicating that impaired immune surveillance contributes significantly to age-related pathologies [19].

### 4.2. Macrophages and Their Interaction with Senescent Cells

Macrophage metabolism plays a key role in senescence-associated multimorbidity by regulating low-grade inflammaging and driving systemic senescence, thus influencing age-related diseases, such as pulmonary fibrosis [47]. The recruitment of macrophages and their interactions with SASP and senescent cells are essential for maintaining tissue homeostasis. Infiltrating and tissue-resident macrophages are key components of the senescent microenvironment, highlighting their crucial role as mediators of immunosenescence [48]. Macrophages can eliminate senescent cells and modulate age-related diseases by promoting inflammation, phagocytosis, efferocytosis, and autophagy, indicating that macrophages have effective regulatory mechanisms in response to senescent microenvironments [49]. Macrophages exhibit significant heterogeneity and plasticity, which are crucial characteristics. Traditionally, their functional heterogeneity has been classified into two groups: M1 (pro-inflammatory) and M2 (immunosuppressive) macrophage polarization [50]. There is a possibility that senescent cells can drive the differentiation of M2 macrophages into three subtypes: M2a, M2b, and M2c, though further research is necessary to confirm this [48]. Additionally, SASP might influence macrophage polarization into other subtypes beyond the M1/M2 classification [50]. Macrophages, recruited by senescent cells, respond to internal and external regulators within senescent microenvironments, influencing neighboring cells and tissues through chemoattraction, inflammaging, fibrosis, and tissue remodeling [19,51].

Recent research has indicated that the interplay between senescent AECs and macrophages may play a role in lung fibrosis [52]. It has been shown that macrophage inhibitory cytokine-1 (MIC-1), secreted by senescent AECs, enhances the activation of M2 macrophages and promotes fibroblast activation in bleomycin-induced lung fibrosis [53]. A recent study revealed that changes in the number and transcriptional identity of alveolar macrophages with aging are not cell autonomous but rather influenced by the alveolar microenvironment, contributing to a senescent phenotype of the immune system response [54]. These findings underscore the potential significance of targeting the senescent lung epithelial microenvironment to restore the functionality of the alveolar macrophage–AEC axis during aging.

### 4.3. B and T Cell Immune Response in a State of Exhaustion

In fibrotic lung diseases, the dynamic process of lung tissue remodeling reflects a bidirectional relationship in which immunosenescence and immunoremodeling mutually influence each other. In the event of a malfunction in the primary epithelial–macrophage defense, a secondary immune response is triggered, led by tissue-resident memory T cells. There is a decrease in the ratio of naïve to memory CD8+ T cells and diminished diversity in the T cell repertoire within both populations [55]. The memory pool expands with the accumulating antigen experience of the host over time, which is probably followed by an increase in B cells. These cells exhibit the loss of surface markers typical of antigen-naïve T cells, including CD28 (recognized as a co-stimulatory molecule essential for activating naïve T cells, which decreases with persistent antigen exposure), while displaying new senescent markers [17,56]. Studies suggest that the increase in memory T cells, followed by an increase in B cells, could result from persistent chronic antigenic stimulation [55,57]. In addition, various studies indicate that CD28− T cells clinically exhibit resistance to immunosuppressants [58]. The exact reasons for dexamethasone resistance in IPF CD28− T cells are unclear, but some transcriptomic data hint at the potential role of reduced expression of the glucocorticoid receptor and HDAC2 transcripts, mirroring findings in CD28− T cells in chronic obstructive pulmonary disease [56]. Additionally, proliferative T cell responses leading to CD28 downregulation are associated with IPF progression, suggesting that assessing circulating CD4 T cells could identify patients prone to deterioration [59]. Nonetheless, despite these insights, the involvement of immune cells and their activation in IPF remains a topic of debate, especially considering the ineffectiveness of immunomodulatory treatments.

In patients with IPF, several reports suggest that adaptive immune responses are frequently chronically stimulated, as demonstrated by the common occurrence of IgG autoantibodies, elevated levels of lymphocyte-derived inflammatory mediators and abnormal levels of T cell activation and clonal proliferation [43,60,61,62,63,64,65]. CD28− T cells show shortened telomeres, express senescence markers, and may exhibit altered effector functions [66]. Activated CD4 T cells, with their singular role in orchestrating adaptive immune responses and their reaction to antigens, may also migrate into lungs affected by IPF. Interestingly, specific changes occur in individuals who have experienced repeated cycles of antigen-driven proliferation, including the loss of CD28 from the cell surface [64]. Therefore, the detection of substantial proportions of circulating CD4+CD28− cells indicates a persistent adaptive immune response, and this capacity could potentially signify an evolutionary adjustment aimed at more efficiently combating resilient antigens. In this regard, some investigations involving IPF patients have revealed a considerable proportion of peripheral CD4+CD28− cells and their manifestation of atypical functional changes that may be pathogenic [59,66]. Notably, significant downregulation of CD28 with its overall cytokine production profile has been associated with poorer clinical outcomes in IPF subjects, consistent with findings in other patient cohorts with chronic immunologic conditions [67,68]. Its reduced secretion of IL-10 could hold significant biological significance in fibrotic conditions, especially considering its potential roles in suppressing harmful immune reactions and inhibiting TGF-β-induced fibrogenesis [69,70,71]. Additionally, along with these exhaustion markers indicating decreased immunofitness in lung fibrosis, there is evidence that accelerated senescence in CD4+ T cells might play a role. Impairment of respiratory chain complex 1 by low-dose rotenone accelerates senescence, highlighting the crucial role of mitochondrial fitness in susceptibility to immunosenescence [72]. Furthermore, cytotoxic CD4+ T cells, often expanded in autoimmune diseases and acute infections like SARS-CoV-2, exhibit a newfound role in senescent fibroblast clearance [73].

Regarding the CD8 T cell compartment, CD8+CD28− T cells, a diverse group comprising memory and exhausted T cells with antigen experience, progressively accumulate with age due to repeated antigen exposure and cytokine signaling. These cells exhibit signs of cellular senescence, such as impaired TCR-mediated proliferation, decreased telomerase activity, and apoptosis resistance [74,75]. Interestingly, during replicative senescence in vitro, CD8+ T cells exhibit a pronounced decrease in CD28 expression, resembling the increase in CD8+CD28− T cells seen in vivo during aging or chronic diseases [66,76]. Conversely, recent findings suggest a complex interplay between CD8+ T cells and senescent cells as they elicit a robust CD8+ T cell-dependent immune response in immunocompetent hosts, potentially mediated by molecular mechanisms such as SASP secretion, enhanced MHC-I expression, damage-associated molecular pattern (DAMP) release, altered antigen presentation, and changes in the immunopeptidome [22,77]. Additionally, there are indications of elevated levels of CD8+ cytotoxic CD28− T cells and increased expression of the immune checkpoint protein PD-1 in IPF lungs, accompanied by a notable elevation in the proportion of structural cells expressing PD-L1 protein compared to healthy lungs [56,78]. This is consistent with the known phenomenon of senescent cells exhibiting heterogeneous expression of programmed death-ligand 1 (PD-L1), with an accumulation of PD-L1+ senescent cells observed with age in vivo [79]. While PD-L1− cells remain susceptible to T cell surveillance, PD-L1+ cells exhibit resistance, suggesting that the heterogeneous expression of PD-L1 has a vital role in the accumulation of senescent cells associated with aging. Moreover, expression of markers of lung T lymphocyte exhaustion is also associated with enhanced TGF-β production and poor survival in IPF [78].

The state of immune aging is also influenced by adaptive alterations in the B cell compartment over the course of fibrosing ILDs. There are deficiencies in B cell precursors, shifts in B cell repertoires, altered B cell dynamics, and decreased immune responsiveness during the aging process. Both intrinsic factors and environmental changes probably contribute to reduced production rates of immature and transitional B cells, with transitional B cells residing longer in their pools and a slower turnover rate in the follicular pool, compensating for the reduced influx of new cells [80]. Interestingly, IPF progression and the occurrence of exacerbations were associated with B cell responses and B cells have the capacity to modify the pro- or anti-fibrotic lung microenvironment, thus influencing fibroblast activity [81,82,83]. Thus, the role of an exhausted B cell compartment in IPF becomes a pertinent question.

### 4.4. NK Cells and NKT-like Cell Contribution to an Aged Immunophenotype

Concerning NK cells, they recognize signals from senescent cells and eliminate them by releasing perforin and granulation enzymes [84]. Senescent fibroblasts upregulate ligands for the activating receptor NKG2D, such as MICA and ULBP2 via endogenous interferon, which enhance NK cell activity [85]. Senescent CD27−CD28−CD8+ T cells express NKG2D and DAP12, activating the cytotoxic function of NK cells and increasing the expression of their corresponding ligands [86]. Therefore, the cytokines released by senescent cells play a crucial role in activating NK cells, indicating an intricate interaction between them. However, the senescent cell characteristics in older individuals might also impede NK cell activation, leading to potential harm to local tissues.

There is still a lack of understanding regarding the phenotype of NK cells in pulmonary fibrosis at the time of diagnosis and how it evolves over time. The literature strongly supports the idea of NK cells functioning as immune senolytics, even outside oncological contexts [22]. Senescent cells increase the expression of the nonclassical MHC class I molecule HLA-E, serving as an inhibitory signal for both CD8 T cells and NK cells. Consequently, depletion of HLA-E renders senescent cells susceptible to elimination by both NK cells and CD8 T cells [30]. Moreover, our group observed abnormalities in circulating immune cells among IPF patients, particularly in the cytotoxic cell domain, including an elevation in NKT-like cells in those with progressive disease, despite antifibrotic therapy. These patients exhibited an overactive and exhausted immunophenotype at diagnosis, which persisted over time [87]. Additionally, we found that NKT-like cells were increased at diagnosis in patients who eventually developed PPF as compared to those with non-PPF, in line with other authors who showed increased NKT-like cells in bronchoalveolar lavage of fibrosing ILDs [42,88]. In the IPF lung, there is a reduced proportion of NK cells with a senescent phenotype and impaired cytotoxic activity [89]. In addition, in vitro experiments with conditioned media from IPF lung fibroblast were shown to induce senescence and reduce the cytotoxicity of healthy NK cells. These results further support the implication of the lung microenvironment in the immune response in the fibrotic lung [65]. Collectively, these findings support the role of dysregulated NK cell responses in lung fibrosis and suggest that immunosenescence may precede and possibly contribute causally to the pathological buildup of senescent cells in chronic diseases like pulmonary fibrosis.

### 4.5. Myeloid-Derived Suppressor Cells and the Immune System–Senescence–Fibrosis Axis

Myeloid-derived suppressor cells (MDSCs) represent a diverse group of immature myeloid cells known for their potent suppressive abilities and their association with adverse outcomes in malignant diseases. They play a significant role in aiding tumor cells to evade immune responses by dampening T cell activity [90,91]. MDSCs lack a standard leukocyte lineage marker classification due to their composition, which includes various immature cells of myeloid origin, such as myeloid-progenitor cells, immature monocytes or dendritic cells, and immature granulocytes. Interestingly, the elevated presence of circulating and tissue-infiltrating MDSCs in IPF patients and other fibrosing ILDs shows an inverse correlation with lung function, suggesting their potential role in the pathogenesis of lung fibrosis [92]. MDSCs and regulatory T cells are recognized for their ability to suppress NK cell cytotoxicity, thereby impeding the elimination of senescent cells from aging tissues [93]. MDSCs do not block the initial steps of T cell activation but instead induce DNA damage and p53 pathway activation in CD8+ T cells through an iNOS-dependent pathway [94]. This suggests a regulatory loop where the accumulation of senescent cells and age-related immunosuppression mutually reinforce each other.

**Figure 2:**
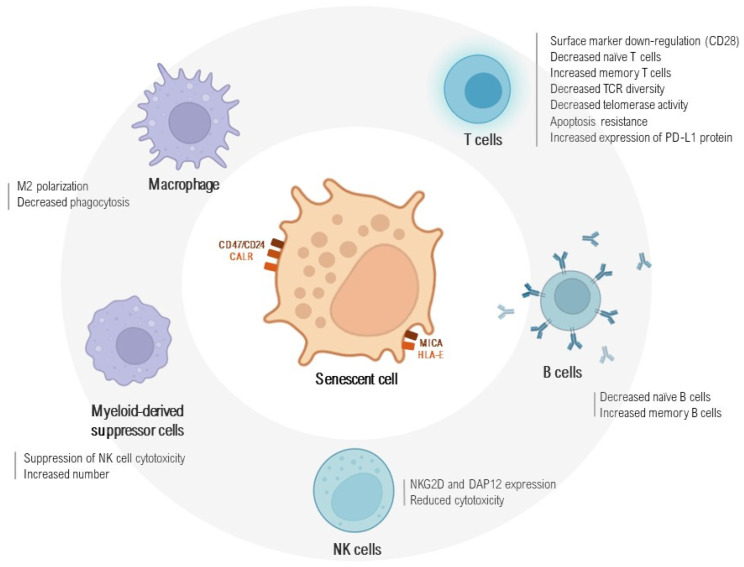
Crosstalk between senescent and immune cells in fibrosing interstitial lung diseases. The biological disturbances in senescent cells enable them to engage in complex communication with the host immune system. Various immune cell populations actively participate in the surveillance and regulation of these senescent cells, highlighting the intricate interplay between cellular senescence and immune responses in pulmonary fibrosis. NK: Natural Killer; NKG2: natural killer group 2; MICA: MHC class I polypeptide-related sequence; HLA-E: Major Histocompatibility Complex; Class I, E, CALR: Calreticulin; CD47: Cluster of Differentiation 47; CD24: Cluster of Differentiation 24. Figure created using BioRender (https://biorender.com), accessed on 7 July 2024.

Figure 2 summarizes the main alterations in senescence-related immune system cells in fibrosing ILDs and their role in the disease process.

## 5. The Potential Link between an Aged Immune System, Autoimmune Disease and Fibrosis

### 5.1. Pulmonary Fibrosis and Autoimmune Features

The intricate relationship between autoimmunity, ILDs and pulmonary fibrosis underscores a complex interplay that warrants exploration. It is widely recognized that several fibrosing ILDs are triggered by autoimmunity, including those linked to conditions like rheumatoid arthritis (RA), systemic sclerosis, primary Sjögren syndrome (pSS), myositis or vasculitis, among others. Furthermore, autoantibodies routinely assessed in clinical contexts are evident across several idiopathic interstitial pneumonias (IIPs) [95]. These serological autoreactivities, such as anti-cyclic citrullinated peptide antibody (anti-CCP), antinuclear antibody (ANA), rheumatoid factor (RF), and anti-neutrophil cytoplasmic antibody, mirror the serologic profiles observed in systemic autoimmune diseases (SAD) [96]. Therefore, the detection of autoantibodies does not equate to autoimmunity, nor does autoimmunity necessarily manifest as an autoimmune disease. These findings may lead to the classification of certain cases as interstitial pneumonia with autoimmune features (IPAF), based on the combination of an IIP with clinical manifestations encompassing pulmonary and extra-thoracic symptoms of an autoimmune disease, serological evidence of autoantibodies and specific imaging patterns, but without fulfilling autoimmune disease criteria [96,97].

### 5.2. Inflammaging and Immunosenescence in Autoimmune Diseases

Since stress resistance mechanisms are crucial for understanding aging in age-related diseases from an evolutionary standpoint, further investigation into their correlation with survival capacity has been undertaken. In summary, insights into immunosenescence suggest that the changes observed over time may be seen as a result of a comprehensive adjustment, where the immune system continuously seeks optimal functioning. Rather than a simple decline, it appears to be an adaptive evolutionary response, possibly extending to stress responses in various tissues. Over time, accumulated stress, including antigenic stress, may impair adaptation, fostering a widespread inflammatory state [98].

Therefore, during the aging process, both senescence and persistent chronic low-grade inflammation (called inflammaging) induce systemic damage to target organs, triggering several alterations that facilitate the onset of age-related chronic diseases. However, recent data are more in line with the notion that the relationship between inflammaging and immunosenescence is not strictly one-way, but rather complementary [17]. The increased release of inflammatory mediators associated with inflammaging reduces the adaptive immune response, eventually leading to immunosenescence. Conversely, the decline in the adaptive immune response further stimulates the innate immune system as a protective mechanism against infections in instances where adaptive immunity is compromised, thereby exacerbating inflammaging [99]. Reduced proteasome function, mitochondrial impairment, dysregulation of control mechanisms, the increased presence of pathogen-associated molecular patterns (PAMPs) and DAMPs, upregulated expression of inflammatory cytokines and accumulation of advanced glycation end products are among the mechanisms contributing to inflammaging and are closely associated with immunosenescence [99,100]. These processes are significant not only as contributors to immune alterations in the elderly but also due to their implications for the development of SAD.

### 5.3. A Tripartite Relationship: Senescence, Autoimmunity, and Lung Fibrosis

Age is regarded as a significant risk factor for a certain degree of autoimmunity, a multifactorial phenomenon that arises from a breakdown in tolerance within the adaptive immune system, resulting in the production of antibodies by plasma cells that target self-antigens [101]. When this leads to tissue damage and excessive inflammation, patients may exhibit symptoms of SAD [102].

The mechanisms that evidence accelerated biological aging in SAD are intricate and must be fully elucidated, and have some shared pathways observed in various fibrosing ILDs. In addition to genetic factors linked to SAD, molecular processes like epigenetic modifications and telomere shortening are intertwined with accelerated biological aging, potentially increasing susceptibility to autoimmune diseases. For instance, patients with RA exhibit greater epigenetic age acceleration compared to healthy counterparts, suggesting that accelerated epigenetic aging may augment the risk and progression of RA [103].

Acknowledging the pivotal role of B cell dysregulation in various autoimmune diseases like systemic lupus erythematosus (SLE), RA, and pSS, it is firmly established that this phenomenon is crucial in both initiating and perpetuating autoimmune diseases, as evidenced by changes in various B cell subsets, including regulatory B cells, memory B cells, and activated B cells, which frequently coincide with disease onset [104,105,106,107]. Nonetheless, the precise mechanisms and implications of impaired B cell tolerance in the progression of disease in SAD-associated pulmonary fibrosis remain incompletely understood. In this regard, telomere length assessment in B cells reveals some abnormalities: naïve and germinal center B cells possess elongated telomeres, while circulating and memory B cells have notably shortened ones [108,109]. Reduced IL-7 production by bone marrow stromal cells impacts B cell quantities and maturation, leading to the emergence of highly autoreactive and pro-inflammatory senescent B cells, ultimately contributing to chronic pulmonary lesions and fibrosis [110,111].

Moreover, regarding T cell malfunction in SAD, premature immunosenescence manifests differently in naïve and effector T cells, leading to the prolonged survival of autoreactive T cells compared to other T cell subsets, including regulatory clones, and it could precipitate cytotoxic autoaggressive damage. Furthermore, the evasion by senescent effector T cells of the regulatory constraints of the standard immune system might instigate cytotoxic activities, resulting in tissue destruction, including lung damage. Lastly, the replicative senescence of regulatory T cells could contribute to deficiencies in anti-inflammatory and anti-autoimmune processes. Other theories suggests that autoimmune conditions are marked by an inflammatory cascade instigated by immune cells targeting tissues, potentially releasing free radicals that could harm telomeres and expedite their degradation, thereby contributing to a self-loop [112]. However, there is currently a lack of studies addressing the whole dynamics of telomere length and telomerase activity in regulatory T cells. Therefore, the telomere/telomerase system serves multifaceted roles, safeguarding genome integrity and modulating cellular aging and lifespan. Notably, in patients with SAD, as well as pulmonary fibrosis, peripheral blood mononuclear cells exhibit shortened telomeres, a phenomenon potentially attributed to chronic psychological stress exposure, heightened inflammation leading to oxidative stress, or deficient telomerase activity [113]. These alterations in the telomere/telomerase system within SAD may imply premature senescence among immune cells and tissue-specific target cells, reflecting the intricate interplay between cellular aging and immune dysregulation in disease progression.

Specifically, the compromised function of peripheral blood CD4+ lymphocytes in RA is believed to influence synovial function and trigger inflammation, marked by reduced proliferation upon stimulation, correlating with shortened telomeres. In addition, and similarly to lung fibrosis, the peripheral blood of RA patients contains enlargement of both memory and CD4+CD28− T cell populations [114]. Interestingly, the reduced expression of α-Klotho, known for its anti-aging and anti-inflammatory properties, in peripheral blood CD4+ cells suggests premature aging potentially linked to decreased CD28 expression, akin to observations in pulmonary fibrosis [115,116]. Similar findings were noted in systemic sclerosis patients, where reduced levels of α-Klotho are linked to a premature aging phenotype rather than solely to the patient’s chronological age [117]. In individuals with SLE, shorter telomeres have been observed relative to healthy counterparts, particularly in whole blood and peripheral mononuclear cells [118,119]. However, the relationship between telomere length and disease activity remains unclear. Therefore, in terms of immune aging, exhausted B and T cells seem to be particularly prone to age-related alterations, mirroring their involvement in SAD, as seen in lung fibrosis contexts.

## 6. Potential Therapeutic Strategies to Improve Responses among Fibrosing Interstitial Lung Diseases

Untreated lung fibrosis inevitably results in premature mortality among all individuals with fibrosing ILDs, and available therapeutic options are limited. There are currently few treatment options available for chronic and progressive interstitial lung diseases, with the two approved drugs, nintedanib and pirfenidone, showing only modest efficacy in slowing the decline of lung function over a one-year period, primarily due to the overwhelming burden of factors involved in the abnormal repair process [4]. Moreover, there is a lack of validated serum biomarker or technique for monitoring disease progression or assessing the fibrosis-induced component within the lung. Current research efforts aim to integrate the natural aging process, immune system and cellular senescence into the understanding of age-related chronic conditions like pulmonary fibrosis. Contemplating senotherapy for fibrosing ILD patients holds particular promise, though several unresolved questions remain. Determining whether the mechanisms underlying SASP and the senescence of epithelial cells, fibroblasts and senescent immune system via antiapoptotic pathways represent definitive targets in lung fibrosis poses a significant challenge. Despite the complexity, pulmonary fibrosis emerges as a promising starting point for conducting proof-of-concept trials involving candidate senolytic drugs or SASP-suppressing agents.

In this context, revitalizing the aged immune system in fibrosing ILD patients through immunotherapy might be promising, but it faces substantial obstacles. The interpretation of immune system changes must be approached with caution as alterations observed with age or health status may reflect adaptations rather than pathological features. While studies have unveiled differences in immune responses between young and old individuals in both innate and adaptive immunity, understanding the mechanisms driving exaggerated and harmful immunosenescence in lung fibrosis requires further exploration [120]. Furthermore, exaggerated and detrimental immunosenescence cannot be accurately measured in clinical practice. It is well established that when the burden of senescent cells surpasses a certain threshold, the self-perpetuating dissemination of senescence via the SASP outpaces the immune system’s ability to clear these cells [121]. Moreover, the increased abundance of SASP factors and a minimum number of transplanted senescent cells may modify immune system function, further exacerbating senescent cell accumulation and inducing accelerated aging-like phenotypes [12,13,122,123,124]. Correspondingly, systemic clearance of senescent cells through genetic or pharmacological approaches tends to mitigate other aging hallmarks and can delay, prevent, or alleviate various age-related disorders and diseases [125,126,127].

**Figure 3:**
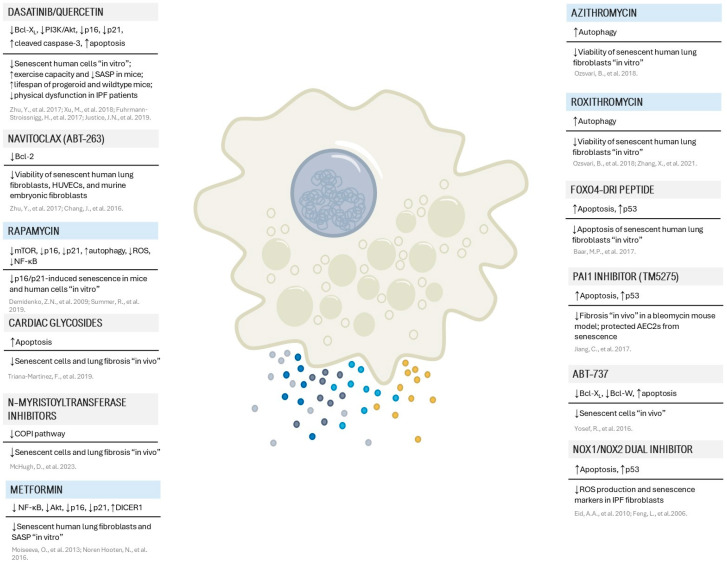
Senolytic strategies with emerging evidence in pulmonary fibrosis. This figure presents a compilation of senolytic treatments validated in lung fibrosis, detailing their mechanisms of action and biological effects. Those highlighted in blue are recognized for their potential to modulate immune pathways [124,125,128,129,130,131,132,133,134,135,136,137,138,139,140,141,142,143].

Pharmacological interventions for senescent cell elimination (so-called senolytics) or attenuation of the detrimental effects of SASP components (senomorphics) aim to disrupt the protective networks and pathways that enable senescent cells to evade apoptosis. In the context of pulmonary fibrosis, senolytic therapies, such as the combination of Dasatinib plus Quercetin, have been identified as inhibiting these protective mechanisms transiently, rendering senescent cells susceptible to apoptosis triggered by their own pro-apoptotic SASP, and also as alleviating physiological dysfunction in IPF patients in the first-in-human clinical trial [21,128]. Interestingly, neither Quercetin nor Dasatinib individually exhibit senolytic properties, but their combination proves senolytic by targeting known factors and also reducing senescence and SASP markers (IL-6, IL8, MCP-1, PAI-1, and GM-CSF) in isolated AEC2s from a bleomycin Ink-Attac mouse model. Several preclinical studies are dedicated to exploring pathways involved in the anti-apoptotic mechanisms of senescent cells, such as BCL-2/BCL-xL, PI3K/AKT, p53/p21/serpins, dependence receptors/tyrosine kinases, and hypoxia-inducible factor-1 pathways. Senotherapy now encompasses various senolytic agents that target key network nodes crucial for shielding senescent cells from apoptosis in pulmonary fibrosis, as outlined in Figure 3.

Despite progress in understanding senescence in lung fibrosis, addressing age-related immune decline remains challenging due to the complex interplay within the immune system and its relationship with fibrosis. Metabolism, interconnected with epigenetic pathways, notably influences immune system aging and cellular senescence. In the aging immune system, sustained trained immune memory may lead to continuous activation even without specific challenges, likely due to shifts in the epigenetic landscape and energy dynamics of innate cells [17]. However, our understanding of the molecular mechanisms underlying immunosenescence in lung fibrosis still needs to be completed, and definitive biomarkers still need to be provided. It is crucial to investigate whether plasma or lung protein profiles linked to the immunosenescence burden can be developed as the accumulation of senescent cells in humans is related to lung damage. Evidence suggests a potential synergy between anti-aging treatments and checkpoint immunotherapy. For instance, combining rapamycin and immunotherapy has shown improved therapeutic outcomes by targeting the SASP [133,134]. Nevertheless, effective immune checkpoint inhibitor therapy in elderly individuals is currently limited, with many therapies still in preclinical or clinical trial stages. Therefore, comprehensive studies on immunosenescence in the context of lung fibrosis are imperative.

## 7. Conclusions

Strong evidence underscores the pathogenic role of cellular senescence in pulmonary fibrosis, offering novel approaches in our battle against fibrosing ILDs. Identifying key pathogenic immune pathways has unveiled potential therapeutic targets that promise to reshape the course of lung fibrosis and potentially enhance survival and quality of life for affected patients. However, the absence of dependable and quantifiable biomarkers for assessing the efficacy of immunosenescence interventions in pulmonary fibrosis related to different etiologies presents a significant hurdle in this domain.

Perhaps, employing drug cocktails to concurrently target multiple pathways holds promise to delay, prevent, or alleviate various age-related disorders such as pulmonary fibrosis. Consequently, further bench research and clinical investigations are warranted to validate and expand upon these promising findings in fibrosing ILDs.
